# The reliability of a linear position transducer for measuring countermovement jump performance in national-level road cyclists

**DOI:** 10.1371/journal.pone.0298291

**Published:** 2024-02-06

**Authors:** Aaron Turner, Mark McKean, Danielle Doyle, Brendan Burkett

**Affiliations:** 1 High-Performance Sport, University of the Sunshine Coast, Sunshine Coast, Sippy Downs, Queensland, Australia; 2 Queensland Academy of Sport, Brisbane, Queensland, Australia; 3 Swimming Australia, Brisbane, Queensland, Australia; Università Telematica degli Studi IUL, ITALY

## Abstract

**Background:**

Jump tests have applications in fatigue monitoring, training intervention evaluations, and talent identification in cycling populations. Due to competitive cycling’s inherent travel demands, linear position transducers (LPTs) are suitable for assessing jump performance in this population as they are a mobile and validated technology. Understanding the reliability and sensitivity of LPTs in assessing jump performance in cycling populations is required to support the development of sports science protocols. Using an LPT, this study aimed to determine the reliability and sensitivity of countermovement jump (CMJ) variables in road cyclists.

**Methods:**

Ten national-level male road cyclists performed three maximal CMJ trials twice per week for two weeks, with an LPT measuring force, power, velocity, repetition rate, vertical distance, and concentric time from each trial. Using the mean and best CMJ results from three trials per testing session, the upper limit 90% confidence interval for the coefficient of variation (CV) and smallest worthwhile change (SWC) determined inter-day and -week reliability (CV ≤ 10%) and sensitivity (acceptable sensitivity = CV ≤ SWC) for CMJ variables.

**Results:**

Mean power and force, velocity (mean and peak), vertical distance (VD), and concentric time had acceptable inter-day and -week reliability when using either the mean or best CMJ results (CV upper limit 90% confidence interval range = 3.54–10.13%). Moreover, the CV and SWC were typically lower when based on the mean rather than the best of three CMJ trials. Lastly, poor sensitivity (CV > SWC) was evident for all CMJ variables.

**Conclusions:**

CMJ-derived mean power and force, velocity (peak and mean), VD, and concentric time have acceptable inter-day and -week reliability when assessed via an LPT in national-level road cyclists. When using an LPT, sports scientists should consider that, while they typically have poor sensitivity, the reliability of CMJ-derived variables improves when expressed as the mean of three trials in national-level road cyclists.

## Introduction

Measuring kinetic and kinematic variables from vertical jump tests is common in sports science, with broad athlete monitoring applications [1–4]. In cycling, evidence supports vertical jump testing in evaluating training interventions [4], differentiating between strong and weak sprint cyclists [5], and monitoring acute fatigue [6]. Moreover, drop jump [6], countermovement jump (CMJ) [5, 7–9], and squat jump [8, 9] performance is strongly related to sprint cycling performance, with the CMJ being the superior protocol for modelling sprint performances (~20 to 30 s) [5, 8], thus supporting historical recommendations for its use in talent identification [10].

Despite motion capture systems and force plates being the criterion technologies for measuring kinetic and kinematic data from the CMJ [11], their practicality for regularly assessing road cyclists during a competition season becomes challenging due to national and international travel demands [12]. Hence, the highly transportable linear position transducer (LPT), a validated technology, is a worthy substitution for assessing CMJ performance [11–16]. However, understanding the underlying reliability of LPTs in assessing CMJ performance is required to help identify suitable outcome measures for monitoring acute fatigue and evaluating training interventions.

Reliability studies assessing athletes from non-cycling populations using LPTs provide some support for CMJ testing in cycling populations [11, 13–15, 17, 18]. Several of these studies indicate that CMJ-derived power [15, 18], force [14, 15, 18], and velocity variables have acceptable reliability [13, 15, 17, 18]. However, the anaerobic power fatigue response to cycling load [6, 19] and the high variation evident in cycling peak power [19, 20], which is strongly related to jump-derived peak power [5, 7], suggests previous CMJ reliability research using LPTs may have reduced applications in cycling athletes. Thus, LPT reliability research based on road cycling athletes is required to support previous and future interventional designs using the CMJ test as an outcome measure in this population.

The number of CMJ trials used (per athlete) in LPT reliability studies varies between research populations, with protocols ranging from two to 10 trials per testing session [11, 13–15, 17, 18]. While these protocols have displayed acceptable reliability for various kinetic and kinematic variables [11, 13–15, 17, 18], CMJ testing protocols for road cycling athletes must consider their time stressors [21] as well as their relatively high training volume [22]. Thus, a pragmatic three-trial protocol for CMJ testing, associated with an improved signal-to-noise ratio (or sensitivity) [23], warrants investigation in road cyclists.

Previous CMJ research has conducted LPT reliability analyses on the mean kinetic and kinematic variables recorded from multiple jump trials [11, 17, 18]. However, athlete monitoring research on road cyclists typically analyses the best performance from multiple jump trials [4, 6, 24]. Consequently, CMJ reliability studies using road cyclists must consider reporting reliability findings based on the mean and best jump performance from multiple trials. Ultimately, this will allow for comparisons to existing CMJ reliability research while improving the application of findings to existing sports science protocols in road cycling.

Aside from reliability, consideration of sensitivity is essential when selecting outcome measures from a given test, as it quantifies the ability to identify a meaningful effect in the presence of measurement variation for a given variable [25]. Since reliability and sensitivity are inseparable [25], researchers report on them collectively for CMJ-derived kinetic and kinematic variables of interest [11, 17, 18, 23, 26]. Ultimately, this enhances the transfer of research to practice, as sports scientists can use the reported reliability and sensitivity information, combined with the expected magnitude of effect (in response to a training intervention), to better select candidate outcome measures from the CMJ test.

Therefore, by assessing in-season CMJ performance in national-level road cyclists with an LPT, the current study primarily aims to determine the inter-day and -week reliability and sensitivity of selected kinetic and kinematic variables. The secondary aim is to compare the reliability and sensitivity of CMJ variables based on the mean and best results recorded from three jump trials per testing session. Satisfying these aims will help sports scientists identify potential CMJ outcome measures for monitoring athletes and evaluating interventions during the cycling season.

## Materials and methods

### Subjects

Ten national-level male road cyclists (age = 22 ± 2 yrs; height = 1.82 ± 0.07 m; body mass = 72 ± 6 kg; functional threshold power = 5 ± 0.33 W/kg) participated in the study, with all athletes representing the same Union Cycliste Internationale Continental cycling team. Each athlete competed in the Australian Cycling National Road Series (the highest domestic racing level) and cycled 16 ± 2 hrs per week over the four weeks before the study commenced. For inclusion in the current study, all athletes had to be apparently healthy and actively competing in the Australian Cycling National Road Series. In contrast, exclusion criteria included those with a current or historical (previous 12 months) musculoskeletal or neurological injury or any medical condition that would likely impair CMJ performance or jeopardise athlete safety by participating in the study.

### Design

To determine the inter-day and -week reliability and sensitivity of CMJ variables recorded with an LPT, participants performed three maximal CMJs twice weekly for two weeks during the second half of the Australian road cycling season.

### Procedures

#### Measurements

Described as the most accurate LPT [27], a GymAware PowerTool (Kinetic Performance Technology, Canberra, Australia) recorded kinetic and kinematic variables from the CMJ test. Variables included force, power, velocity, concentric time, repetition rate, and vertical distance, with the latter described as the vertical distance travelled from the start to the end of the concentric phase, calculated via basic trigonometry using the LPTs tether and angle sensor (Table 1) [28].

**Table 1:** Kinetic and kinematic variables derived from the countermovement jump.

	Description	Expression
Vertical distance (m)	The distance covered from the start to the end of the concentric phase	Total
Force (N)	The force applied during the concentric phase	Mean and peak
Power (W & W/kg)	The absolute and relative power applied during the concentric phase	Mean and peak
Velocity (m/s)	The velocity recorded during the concentric phase	Mean and peak
Concentric time (s)	Duration of the concentric phase	Total
Repetition rate (n/min)	The rate of repetition performance over one minute based on the duration of a single repetition	Rate

#### Testing

As per previous research [15, 17], testing sessions included the LPT (zeroed before each session) attached to a wooden dowel (weight = 400 g, length = 1.35 m), held in the back squat position while performing three maximal CMJs. Specifically, with the LPT tether attached five centimetres from the right end of the dowel (relative to the athlete) and the LPT unit magnetically mounted to a weight plate on the ground below, the following verbal instructions preceded the jump performance: “When you are ready, please perform three maximal countermovement jumps, ensuring you reset after each attempt.” Subsequently, athletes performed a rapid eccentric squat followed by a rapid concentric squat, resulting in a jump, then landing via an eccentric squat and returning to the start position (i.e., standing upright). Intertrial rest was ~3 s, with maximal attempts preceded by a standardised warm-up consisting of:

- Lower-body foam rolling (one minute each on posterior shanks, anterior thighs, posterior thighs, and lateral thighs)
- Dynamic mobility exercises for one set of ten repetitions each (supine arm raises, supine hamstring floss, trunk rotations)
- Isotonic exercises for two sets of eight repetitions (unloaded squats, unloaded standing calf raises)
- Test specific preparation movements for one set of three repetitions (submaximal CMJ)

While the warm-up was specific to the present population and testing procedure, the combination of lower-body foam rolling, dynamic stretching, and unloaded isotonic exercises are recommended warm-ups with demonstrated performance benefits in young adults [29].

Testing sessions were completed on Tuesday and Thursday (PM; 1530–1600) for two weeks (four testing sessions in total), with the mean and best CMJ results per session and athlete used in statistical analyses.

Lastly, athletes undertook three familiarisation sessions that replicated the testing protocol two weeks before the primary data collection phase. This inclusion allowed for technical corrections and feedback as required to improve CMJ performance, which was absent during the primary data collection phase.

### Statistical analysis

Using RStudio (Build 524, “Mountain Hydrangea” Release, R version 4.3.0) and a significance threshold of α = 0.05, analysis and calculation of descriptive statistics and 90% confidence intervals (CIs) for relevant variables were completed.

### Data review and preparation

The mean and best performances from three CMJ trials per testing session were extracted from the original dataset, resulting in two separate datasets to analyse. Upon completing visual (histograms and density plots) and statistical (Shapiro-Wilk test) normality assessments for each dataset [30], the current study employed a nonparametric statistical methodology.

The Friedman test determined if CMJ results were significantly different between testing sessions, with subsequent findings (P-value > 0.05) warranting the calculation of nonparametric bootstrapped 90%CIs (bias-corrected and accelerated; resamples with replacement = 999) for reliability and sensitivity variables [31, 32], using pooled CMJ data [33].

#### Inter-day reliability

The coefficient of variation (CV) determined the reliability of CMJ variables, calculated as (*TE/mean*)×100, with *mean* representing the mean of all trial data from Tuesday and Thursday testing sessions for a given week [34, 35]. The *TE*, calculated as *SD_diff_*/√2, represented the typical error, with *SD_diff_* calculated as the standard deviation (SD) of the difference in results between the Tuesday and Thursday testing sessions [34, 35]. As this method resulted in the calculation of two TE and CV values per CMJ variable (one for each week of the study design), the respective means were reported for the inter-day period. Using the upper limit of 90%CIs, acceptable reliability described CMJ variables with a CV ≤ 10% [14, 18, 26].

#### Inter-week reliability

Inter-week reliability calculations were the same as inter-day reliability. However, the TE and CV calculations used results from weekly testing sessions (i.e., Tuesday versus Tuesday and Thursday versus Thursday).

#### Sensitivity

The inter-day and -week smallest worthwhile change (SWC = 0.20×*between subject SD of baseline*) for a given CMJ variable was expressed as a percentage of the mean of the baseline results [36]. As this method resulted in the calculation of two SWC values per CMJ variable, due to the multi-week study design, the respective mean SWCs were reported for each period (inter-day and -week). Using their respective upper limit of 90%CIs, acceptable sensitivity described CMJ variables with a CV ≤ SWC [17, 18, 26].

## Results

### Testing session comparisons

**Fig 1:**
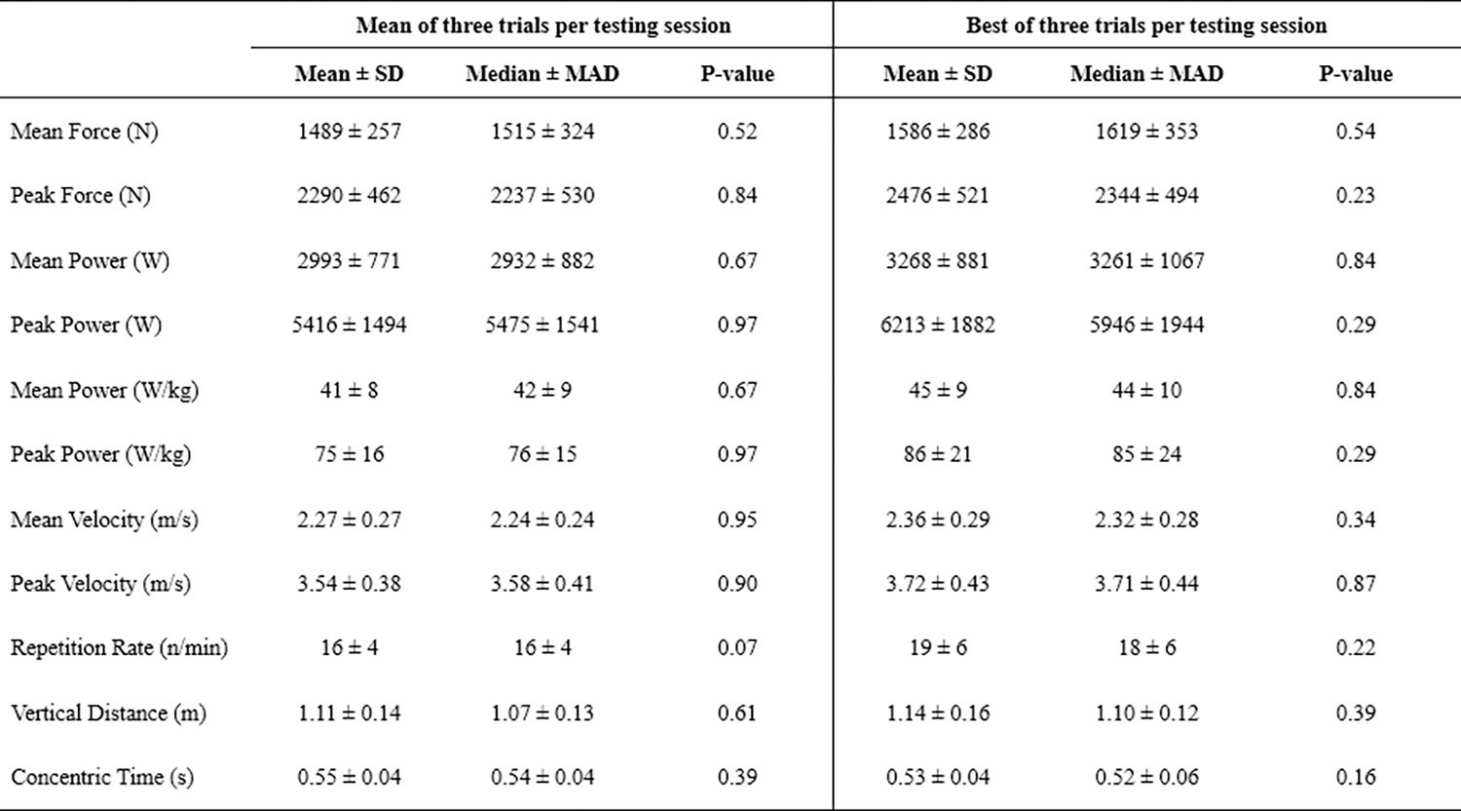
Descriptive statistics, using pooled data, for selected kinetic and kinematic variables from the countermovement jump. SD = standard deviation; MAD = median absolute deviation; P-value derived from the Friedman test comparing results from the four testing sessions.

The Friedman test indicated no effect of testing session on CMJ results for either the mean or best of three trials, as evidenced by the non-significant P-values (range = 0.07–0.97, Fig 1).

### Inter-day reliability

**Fig 2:**
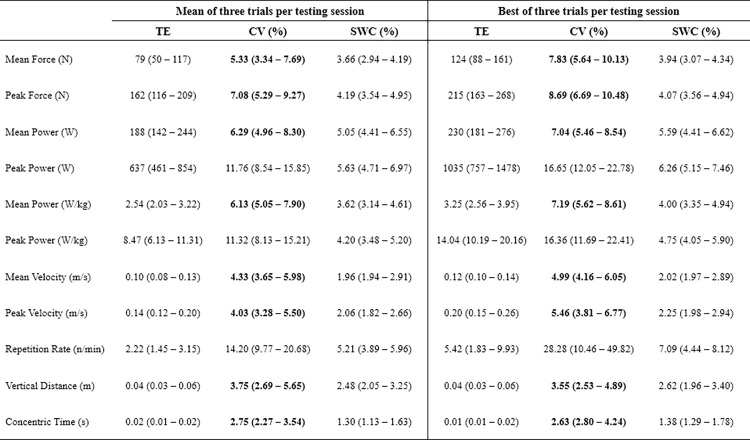
Inter-day reliability of selected kinetic and kinematic variables from the countermovement jump. TE = typical error; CV = coefficient of variation; SWC = smallest worthwhile change; values in bold are those displaying 90% confidence intervals (CI) with acceptable reliability (upper limit of 90%CI ≤ 10%).

**Fig 3:**
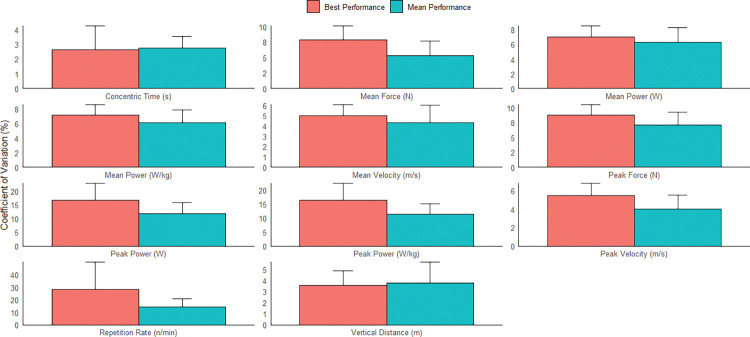
Column chart contrasting the inter-day reliability of the best and mean CMJ performance. Y-error bars represent the 90% confidence interval upper limit for each variable.

Analysis indicated that concentric time, vertical distance, velocity (mean and peak), force (mean and peak), and mean power (absolute and relative) had acceptable inter-day reliability when using either the mean or best of three CMJ trials (CV upper limit of 90%CI range = 3.54–10.48%, Figs 2 and 3). In contrast, peak power (absolute and relative) and repetition rate did not display acceptable inter-day reliability (CV upper limit of 90%CI > 10%). Lastly, the TE, CV, and SWC were typically lower when based on the mean rather than the best of three CMJ trials.

### Inter-week reliability

**Fig 4:**
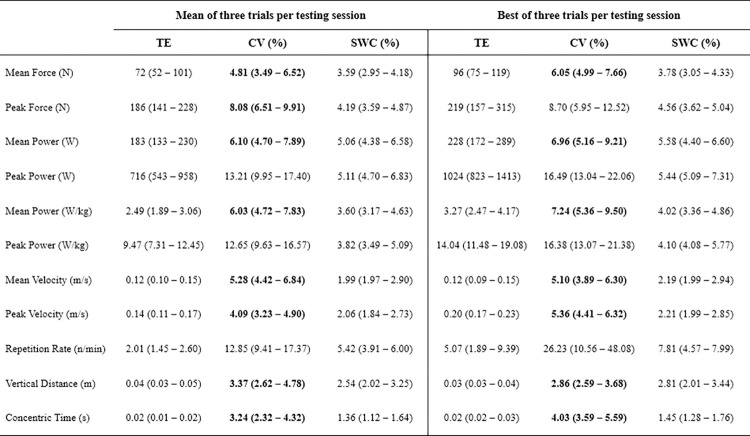
Inter-week reliability of selected kinetic and kinematic variables from the countermovement jump. TE = typical error; CV = coefficient of variation; SWC = smallest worthwhile change; values in bold are those displaying 90% confidence intervals (CI) with acceptable reliability (upper limit of 90%CI ≤ 10%).

**Fig 5:**
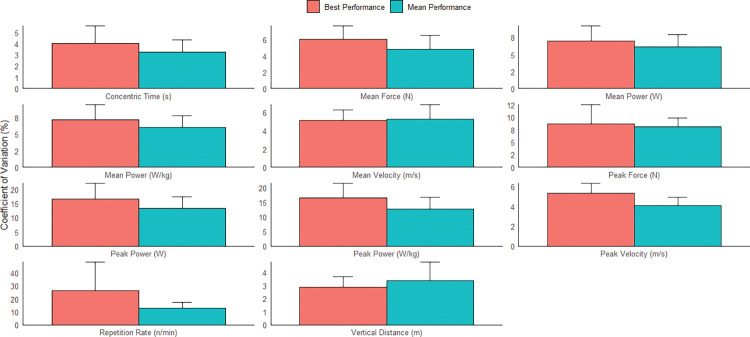
Column chart contrasting the inter-week reliability of the best and mean CMJ performance. Y-error bars represent the 90% confidence interval upper limit for each variable.

Concentric time, vertical distance, velocity (mean and peak), mean force, and mean power (absolute and relative) displayed acceptable inter-week reliability when using either the mean or best of three jump trials (CV upper limit of 90%CI range = 3.68–9.50%, Figs 4 and 5). Interestingly, peak force only had acceptable inter-week reliability when using the mean of three trials. Lastly, as per inter-day reliability results, the TE, CV, and SWC were typically lower when based on the mean of three CMJ trials.

### Sensitivity

No CMJ variable displayed acceptable inter-day or -week sensitivity (Figs 2 and 4).

## Discussion

The current study used an LPT to determine the in-season inter-day and -week reliability and sensitivity of CMJ-derived kinetic and kinematic variables in national-level road cyclists, using the mean and best results recorded from three jump trials per testing session.

### Reliability

Reliability calculations based on the mean and best CMJ performance from three trials yielded similar inter-day and -week reliability. Despite the similar number of CMJ variables achieving acceptable inter-day and -week reliability regardless of whether comparing best versus mean performance, the CV point estimates and 90%CIs calculated from mean CMJ performance were typically lower. This finding is consistent with previous CMJ research using force plates, highlighting that mean performance for jump height and peak velocity, force, and power has a lower CV than the best performance when using a three-trial protocol [23]. Based on the current and previous findings [23], sports scientists seeking to reduce variation and increase the reliability of CMJ variables should consider using the mean performance from multiple trials in practice. Moreover, the aforementioned approach would improve the sensitivity of a given variable when the SWC is a pre-determined fixed value (e.g., 5%).

The current reliability findings are similar to previous CMJ research using LPTs with field- and court-based athletes, regardless of whether comparing point estimates or 90%CIs [13, 17, 18]. Specifically, using the mean of six trials per testing session, CMJ results from field-based athletes also highlight that force (mean and peak) [18], mean power (absolute) [18], and peak velocity [17, 18] have acceptable inter-week reliability. In addition, previous findings from CMJ research using an LPT with court-based athletes highlighted that velocity (mean and peak) has acceptable inter-day reliability when using the best performance from three trials [13], consistent with the current study’s cycling cohort findings.

Unlike field-based athletes [18], peak power (absolute and relative) did not display acceptable inter-week reliability in the current population when using the mean of three CMJ trials. This contrasting finding is unsurprising in a cycling population due to the fatigability of anaerobic power in response to cycling load [6, 19], resulting in high variations in cycling peak power [19, 20], which is strongly related to vertical jump performance [7–9], including jump-derived peak power [7]. This concept partly explains why peak power measures displayed poor inter-day and -week reliability in the current study. Thus, in practice, CMJ-derived mean power is a more suitable outcome measure in cycling populations due to its acceptable inter-day and -week reliability, attributable to the smoothing function of the mean [37].

As mentioned, velocity variables were reliable in the current study, displaying comparatively low inter-day and -week CVs compared to other variables, consistent with CMJ research using LPTs with field- and court-based athletes [13, 18]. Naturally, the reliability of velocity measures was paralleled by low variability in their constituents, vertical distance and concentric time. The reason velocity variables are more reliable than peak power variables in the current study may result from athlete movement compensations [38, 39]. Specifically, in the presence of varying training loads, which CMJ peak power is susceptible to [39–41], an athlete may compensate for power-demanding tasks by changing their force production [39], ultimately increasing power and force variability between testing sessions [39]. Hence, compared to peak velocity, such compensations may partly explain the greater inter-day and -week variability in peak power and force identified in the current study.

### Sensitivity

Sensitivity interacts with reliability, with the latter describing measurement variation or error (i.e., TE and CV) and the former describing the ability to detect the SWC in the presence of measurement variation [25]. A combined understanding of sensitivity and reliability aids variable selection for sports science interventions and the interpretation of findings [36]. Therefore, it was prudent to report measurement sensitivity alongside reliability to improve research applications and maintain cohesion with previous CMJ literature using LPTs [11, 17, 18] and force plates [23, 26].

The sensitivity of CMJ-derived variables using LPTs varies between the previous and current findings. Upon reviewing inter-week findings from field-based athletes, point estimation of peak velocity (mean of six trials) displays acceptable sensitivity [17, 18]. However, these previous sensitivity findings involved a larger SWC and twice as many CMJ trials per session compared to the current study [17, 18], ultimately increasing the likelihood of detecting the SWC in the presence of measurement variation [23]. Sports scientists must consider the practicality of increasing the SWC or the number of CMJ trials per session if implementing testing as an outcome measure (what is the expected magnitude of change?) or fatigue monitoring protocol (is there enough time?), respectively.

Interestingly, the sensitivity of CMJ variables assessed in field-based athletes appears to be impacted by diurnal variation [18]. Specifically, when using an LPT with field-based athletes, point estimation of jump height and peak velocity and power (mean of six trials) have lower sensitivity (or a reduced ability to detect the SWC) when CMJ testing occurs in the afternoon rather than the morning [18]. Thus, previously reported results from an afternoon CMJ testing protocol [18], akin to the current study’s protocol, support the poor sensitivity findings associated with power (peak and mean) and peak velocity evident in the current study. Further research is required to determine if the same diurnal variation in sensitivity for CMJ variables is evident in cycling populations.

Variables with high sensitivity are ideal because a genuine improvement (or decline) in performance is, at a minimum, a performance change exceeding the SWC by a magnitude equivalent to the TE (or CV) [36]. Although no CMJ variables investigated in the current study had acceptable sensitivity, it is essential to note that poor sensitivity does not automatically prohibit their use in interventional designs [42, 43]. This notion arises because a variable with a very low CV (high reliability) and acceptable sensitivity may be inherently unresponsive to a training intervention or training load in general [18]. In contrast, the SWC may easily be detected in a variable with a high CV (low reliability) if it is highly responsive to an intervention [43]. In the latter scenario, a variable with poor sensitivity would still be a worthy outcome measure. Therefore, in combination with the underlying sensitivity and reliability, researchers should carefully consider the expected magnitude of change, or effect, when selecting CMJ variables as outcome measures in a cycling population.

### Methodology

Measures of uncertainty determined the reliability and sensitivity of CMJ variables rather than point estimates, with 90%CIs preferentially used to maintain consistency with previous CMJ research using LPTs [11, 13, 17]. Had point estimates guided the current interpretations, no additional variables would have achieved acceptable sensitivity; however, inter-week peak force (based on best CMJ performance) would have been deemed reliable. Our interpretations and recommendations based on uncertainty stemmed from the concept that population-level reliability and sensitivity estimates, rather than sample-level estimates, are more desirable [44]. Future research should consider basing their recommendations on measurement uncertainty rather than point estimates, ultimately aiding the generalisability of their reliability and sensitivity findings [45].

Increasing sample size does improve the precision of factor-generated CIs, which has a reverse utility, where desired precision can determine the sample size required in reliability studies [35]. However, the sample size used in the current study was constrained to the size of the cycling team recruited. While the use of bootstrapped CIs increased the generalisability of the current findings, future reliability research using much larger sample sizes will be able to provide improved precision.

The small size and weight of LPTs ensure they are well suited to the demands of high-performance sports, including national and international travel requirements [12]. Moreover, the inherent validity of the units further supports their continued use in CMJ testing [11–16]. However, due to some reported measurement biases with LPTs during CMJ testing [12, 15, 16], results cannot be interchanged with or interpreted against non-LPT testing devices [12, 15, 16]. Consequently, the current paper has primarily confined research comparisons to those that have employed LPTs in their CMJ testing and recommends that the current findings be considered in training environments that use an LPT with cycling populations.

Among the research employing LPTs for their CMJ testing, attachment sites of the associated tether include either the participant’s waist [11, 13–15] or a bar or dowel held in the back squat position [15, 17, 18]. Although the current study used the latter protocol, both attachment sites produce similar CMJ results and reliability [15]. Therefore, comparisons between the current and previous LPT research using different attachment sites for CMJ testing were considered appropriate.

Unlike team sports such as netball, handball, volleyball, and Australian Rules Football, road cycling does not involve jumping and landing skills akin to the CMJ. This inherent lack of CMJ experience is partly evident in CMJ performance comparisons among Norwegian national team representatives, with road cyclists achieving one of the lowest mean CMJ heights among 44 sports [46]. While the current study’s familiarisation period involved CMJ technical cues to improve subsequent jump performance, the current samples’ comparatively low exposure to CMJ training unlikely introduced another source of variability. In support, LPT research from non-athletic populations highlights that the inter-week reliability of jump height (CV = 5.3–6%) and peak velocity (CV = 4.5–5.2%) [15] is similar to that of Australian Rules Football players (jump height CV = 6.6%, peak velocity CV = 6.8%) [17]. This finding is consistent with force plate research highlighting that the CV for jump height is not statistically different between sedentary and active (> 6 hours of sport per week) adolescents [47]. Lastly, participant age, rather than activity experience, appears more detrimental to intra- and inter-session CMJ reliability [48, 49], with improved reliability evident in teenagers and adults [48, 49], a demographic captured in the current study.

While using a consistent CMJ testing environment, the current study’s primary data collection occurred during the cycling season, ultimately capturing valuable reliability data during a period associated with stressors such as travel and competition [50]. Aside from being the most prolonged training period, and thus arguably the most relevant, for road cyclists [51], the decision to collect CMJ data during the cycling season avoided the high variability in training loads associated with preseason training [51] and the inherent injury risks [52, 53]. Given the rationale for our methodological decision, attention inadvertently shifts to whether CMJ inter-day and -week reliability differs significantly between preseason and competition season. Previous studies highlight that the intra-session reliability of CMJ variables is not dissimilar when comparing pre-, mid-, and post-season results in team sports [38, 54, 55], with similar in-season stressors [56–58], despite the more pronounced training load-induced fatigue evident in preseason CMJ results [59]. With further research required to determine if these team sport findings [54, 55] are ubiquitous for inter-day and -week CMJ reliability in cycling populations, the current findings are most applicable to in-season CMJ protocols for cyclists when considering the data collection timing.

### Limitations

The current study is not without limitations, with the first being that the reliability findings are from a representative sample. While reporting CIs improves the current study’s generalisability, the application can improve by considering the study context (competition season) and cycling demographic. Where possible, sports scientists should conduct an independent reliability analysis on CMJ variables collected from their athletes to improve variable selection and the detection of performance changes. Secondly, using a loaded CMJ condition (40 kg), inter-week reliability improves when testing sessions occur in the morning [18]. While this may have improved reliability in the present study’s unloaded CMJ conditions, it was impractical due to the athletes undertaking their cycling training each morning. Thirdly, with some CMJ literature using six to 10 CMJ trials per testing session [15, 17, 18], the three-trial protocol used in the current study may appear as a limitation. However, given the demand for time-economic testing procedures [60] and the acceptable reliability [26] and sensitivity [23] of single- and three-trial CMJ protocols, respectively, the three-trial protocol was considered pragmatic. Lastly, pre-testing warm-up modes and durations vary in LPT-based CMJ reliability research [11, 13–15, 18]. Different combinations of running, cycling, jumping, balancing, resistance training, and stretching are evident in warm-ups taking ~ 10 mins [11, 13–15, 18]. The current study did not include aerobic exercises in the warm-up due to the relatively high training volumes undertaken by road cyclists [22]. Instead, jump-specific movement patterns following foam rolling and dynamic stretching were preferentially used [29], with the latter improving joint range of motion without impacting jump performance [61, 62].

### Practical applications

Given the anaerobic power fatigue response to cycling load [6, 19] and the high variation in cycling peak power [19, 20], which is strongly related to jump-derived peak power [5, 7], previous CMJ reliability research using LPTs had reduced applications in cycling athletes. Therefore, the current study contributes to the existing LPT-based research [11, 13–15, 17, 18] by providing CMJ reliability point estimates and uncertainty specific to a cycling population. Sports scientists can use the current findings to select appropriate CMJ-derived outcome measures to evaluate and interpret the effect of a training intervention. Moreover, for athlete monitoring protocols employing the CMJ test to determine neuromuscular fatigue (or athlete readiness), the current reliability and SWC values aid sports scientists in determining meaningful changes in CMJ performance [35, 36]. Lastly, the current reliability and SWC values provide a basis for sample size planning in interventional research [63], with the minimally important difference (a genuine performance change) in CMJ performance being the sum of the SWC and TE (or CV) for a given variable [36].

## Conclusions

Using an LPT to assess in-season CMJ performance in national-level road cyclists, the current study identified acceptable inter-day and -week reliability for mean power (absolute and relative), velocity (mean and peak), mean force, concentric time, and vertical distance. Broadly, when using an LPT, the reliability of CMJ variables improves when using the mean performance from three CMJ trials rather than the best. When using the CMJ as an intervention outcome measure, sports scientists should consider that CMJ variables typically have poor sensitivity when assessed with an LPT. Lastly, the current findings have applications in evaluating and interpreting training interventions, neuromuscular fatigue monitoring, and research sample size planning when using the CMJ test as an outcome measure in a road cycling population.

## Supporting information

S1 Table Mean countermovement jump performances from national-level road cyclists. (DOCX)

S2 Table Best countermovement jump performances from national-level road cyclists. (DOCX)
