# Supplementary material for: The reliability of a linear position transducer for measuring countermovement jump performance in national-level road cyclists
Source: PLoS One. 2024 Feb 6;19(2):e0298291. doi: 10.1371/journal.pone.0298291 (PMC10846725; doi:10.1371/journal.pone.0298291)
Supplement: S1 Table — (DOCX) [file pone.0298291.s001.docx]

| **S1 Table: Mean countermovement jump performances from national-level road cyclists** | | | | | | | | | | | | | |
| --- | --- | --- | --- | --- | --- | --- | --- | --- | --- | --- | --- | --- | --- |
| **Athlete ID** | **Mean Force (N)** | **Peak Force (N)** | **Mean Power (W)** | **Peak Power (W)** | **Mean Power (W/kg)** | **Peak Power (W/kg)** | **Mean Velocity (m/s)** | **Peak Velocity (m/s)** | **Rep Rate (n/min)** | **Vertical Distance (m)** | **Conc. Time (s)** | **Day #** | **Week #** |
| 1 | 1284 | 1888 | 2261 | 4262 | 33.252 | 62.673 | 1.985 | 3.137 | 17.658 | 0.961 | 0.53 | 2 | 1 |
| 1 | 1284 | 1871 | 2325 | 4469 | 34.198 | 65.718 | 2.045 | 3.226 | 14.534 | 1.002 | 0.537 | 4 | 1 |
| 1 | 1298 | 1741 | 2219 | 4567 | 32.629 | 67.157 | 1.862 | 3.152 | 16.014 | 0.925 | 0.537 | 2 | 2 |
| 1 | 1383 | 1793 | 2348 | 4569 | 34.528 | 67.189 | 1.817 | 3.191 | 14.072 | 0.953 | 0.544 | 4 | 2 |
| 2 | 1363 | 1949 | 2691 | 4423 | 42.043 | 69.112 | 2.250 | 3.633 | 20.440 | 1.026 | 0.497 | 2 | 1 |
| 2 | 1269 | 1981 | 2411 | 4595 | 37.676 | 71.800 | 2.208 | 3.292 | 17.360 | 0.967 | 0.490 | 4 | 1 |
| 2 | 1260 | 2006 | 2542 | 4452 | 39.726 | 69.56 | 2.355 | 3.421 | 19.859 | 1.066 | 0.510 | 2 | 2 |
| 2 | 1367 | 2340 | 2730 | 4593 | 42.655 | 71.766 | 2.277 | 3.384 | 17.804 | 0.915 | 0.464 | 4 | 2 |
| 3 | 1602 | 2410 | 3128 | 6975 | 41.371 | 92.261 | 2.177 | 3.643 | 18.194 | 1.043 | 0.516 | 2 | 1 |
| 3 | 1557 | 2454 | 2873 | 4449 | 38.009 | 58.846 | 2.132 | 3.268 | 18.471 | 1.037 | 0.536 | 4 | 1 |
| 3 | 1522 | 2326 | 2964 | 4613 | 39.212 | 61.021 | 2.216 | 3.383 | 20.182 | 1.027 | 0.538 | 2 | 2 |
| 3 | 1549 | 2308 | 2872 | 5909 | 37.986 | 78.164 | 2.031 | 3.374 | 17.861 | 1.020 | 0.550 | 4 | 2 |
| 4 | 1163 | 1725 | 2085 | 3471 | 32.079 | 53.399 | 2.026 | 3.064 | 11.105 | 1.032 | 0.551 | 2 | 1 |
| 4 | 1209 | 1729 | 2255 | 3962 | 34.693 | 60.956 | 2.071 | 3.384 | 16.379 | 1.091 | 0.571 | 4 | 1 |
| 4 | 1206 | 1789 | 2272 | 3898 | 34.957 | 59.966 | 2.136 | 3.258 | 15.042 | 1.075 | 0.545 | 2 | 2 |
| 4 | 1159 | 1640 | 2076 | 3280 | 31.937 | 50.466 | 2.015 | 3.150 | 16.094 | 1.043 | 0.550 | 4 | 2 |
| 5 | 1584 | 2182 | 3233 | 6015 | 44.053 | 81.949 | 2.280 | 3.828 | 18.612 | 1.091 | 0.536 | 2 | 1 |
| 5 | 1619 | 2781 | 3382 | 5527 | 46.074 | 75.305 | 2.489 | 3.620 | 18.591 | 1.075 | 0.502 | 4 | 1 |
| 5 | 1594 | 2310 | 3142 | 6190 | 42.809 | 84.337 | 2.222 | 3.705 | 17.283 | 1.111 | 0.543 | 2 | 2 |
| 5 | 1458 | 2298 | 2899 | 6020 | 39.493 | 82.022 | 2.268 | 3.597 | 16.495 | 1.110 | 0.564 | 4 | 2 |
| 6 | 2019 | 3077 | 4007 | 6661 | 51.374 | 85.397 | 2.351 | 3.793 | 23.903 | 1.217 | 0.570 | 2 | 1 |
| 6 | 1658 | 3033 | 3753 | 6120 | 48.109 | 78.458 | 2.709 | 3.763 | 21.811 | 1.254 | 0.557 | 4 | 1 |
| 6 | 1715 | 2838 | 3861 | 6791 | 49.498 | 87.069 | 2.747 | 4.006 | 21.984 | 1.263 | 0.544 | 2 | 2 |
| 6 | 1735 | 3008 | 4067 | 8119 | 52.145 | 104.09 | 2.859 | 4.150 | 17.143 | 1.261 | 0.530 | 4 | 2 |
| 7 | 1663 | 2265 | 3243 | 5965 | 46.329 | 85.216 | 2.166 | 3.567 | 13.711 | 0.952 | 0.497 | 2 | 1 |
| 7 | 1747 | 2202 | 3267 | 5940 | 46.671 | 84.862 | 2.079 | 3.583 | 11.652 | 0.975 | 0.496 | 4 | 1 |
| 7 | 1660 | 2442 | 3327 | 5423 | 47.533 | 77.476 | 2.315 | 3.404 | 14.956 | 0.968 | 0.498 | 2 | 2 |
| 7 | 1746 | 2700 | 3560 | 6664 | 50.854 | 95.196 | 2.312 | 3.718 | 13.377 | 0.993 | 0.483 | 4 | 2 |
| 8 | 1162 | 1852 | 2161 | 3807 | 32.796 | 57.765 | 2.100 | 3.102 | 14.338 | 1.096 | 0.543 | 2 | 1 |
| 8 | 1077 | 1809 | 1878 | 3329 | 28.492 | 50.517 | 1.953 | 2.928 | 13.436 | 1.060 | 0.583 | 4 | 1 |
| 8 | 1053 | 1742 | 1760 | 3180 | 26.707 | 48.254 | 1.904 | 2.898 | 10.686 | 1.052 | 0.605 | 2 | 2 |
| 8 | 1086 | 2081 | 1900 | 2818 | 28.838 | 42.762 | 1.99 | 2.885 | 13.986 | 1.076 | 0.577 | 4 | 2 |
| 9 | 1784 | 2790 | 3910 | 7091 | 47.105 | 85.432 | 2.454 | 3.842 | 18.661 | 1.310 | 0.594 | 2 | 1 |
| 9 | 1881 | 3183 | 4666 | 7955 | 56.219 | 95.845 | 2.812 | 4.335 | 8.385 | 1.481 | 0.611 | 4 | 1 |
| 9 | 1932 | 3343 | 4504 | 8963 | 54.267 | 107.986 | 2.742 | 4.249 | 11.971 | 1.461 | 0.617 | 2 | 2 |
| 9 | 1912 | 2908 | 4610 | 7762 | 55.541 | 93.513 | 2.818 | 4.319 | 14.059 | 1.420 | 0.584 | 4 | 2 |
| 10 | 1465 | 2125 | 3030 | 6809 | 41.964 | 94.314 | 2.315 | 3.868 | 9.804 | 1.213 | 0.578 | 2 | 1 |
| 10 | 1508 | 2149 | 3309 | 6044 | 45.828 | 83.705 | 2.507 | 3.920 | 9.993 | 1.278 | 0.584 | 4 | 1 |
| 10 | 1422 | 2209 | 2890 | 5563 | 40.027 | 77.045 | 2.305 | 3.717 | 10.021 | 1.172 | 0.578 | 2 | 2 |
| 10 | 1612 | 2339 | 3286 | 5390 | 45.506 | 74.652 | 2.321 | 3.681 | 8.422 | 1.163 | 0.577 | 4 | 2 |
