# Supplementary material for: The reliability of a linear position transducer for measuring countermovement jump performance in national-level road cyclists
Source: PLoS One. 2024 Feb 6;19(2):e0298291. doi: 10.1371/journal.pone.0298291 (PMC10846725; doi:10.1371/journal.pone.0298291)
Supplement: S2 Table — (DOCX) [file pone.0298291.s002.docx]

| **S2 Table: Best countermovement jump performances from national-level road cyclists** | | | | | | | | | | | | | |
| --- | --- | --- | --- | --- | --- | --- | --- | --- | --- | --- | --- | --- | --- |
| **Athlete ID** | **Mean Force (N)** | **Peak Force (N)** | **Mean Power (W)** | **Peak Power (W)** | **Mean Power (W/kg)** | **Peak Power (W/kg)** | **Mean Velocity (m/s)** | **Peak Velocity (m/s)** | **Rep Rate (n/min)** | **Vertical Distance (m)** | **Conc. Time (s)** | **Day #** | **Week #** |
| 1 | 1320 | 2027 | 2312 | 4793 | 33.994 | 70.488 | 2.073 | 3.222 | 18.597 | 0.989 | 0.503 | 2 | 1 |
| 1 | 1326 | 1900 | 2484 | 4732 | 36.531 | 69.583 | 2.176 | 3.36 | 15.675 | 1.016 | 0.524 | 4 | 1 |
| 1 | 1435 | 1943 | 2656 | 5981 | 39.058 | 87.957 | 1.966 | 3.615 | 16.624 | 0.967 | 0.523 | 2 | 2 |
| 1 | 1456 | 1876 | 2486 | 5165 | 36.566 | 75.951 | 2.007 | 3.454 | 15.984 | 0.956 | 0.524 | 4 | 2 |
| 2 | 1421 | 2034 | 2842 | 5094 | 44.411 | 79.589 | 2.281 | 3.717 | 22.784 | 1.040 | 0.483 | 2 | 1 |
| 2 | 1342 | 2144 | 2530 | 4640 | 39.535 | 72.505 | 2.324 | 3.327 | 21.960 | 0.974 | 0.483 | 4 | 1 |
| 2 | 1316 | 2066 | 2794 | 4933 | 43.660 | 77.078 | 2.505 | 3.493 | 23.360 | 1.100 | 0.484 | 2 | 2 |
| 2 | 1485 | 2467 | 2962 | 5361 | 46.283 | 83.772 | 2.434 | 3.451 | 20.767 | 0.943 | 0.443 | 4 | 2 |
| 3 | 1644 | 2730 | 3302 | 8568 | 43.678 | 113.331 | 2.257 | 3.765 | 21.277 | 1.094 | 0.503 | 2 | 1 |
| 3 | 1637 | 2602 | 2968 | 4916 | 39.256 | 65.032 | 2.182 | 3.339 | 23.050 | 1.048 | 0.523 | 4 | 1 |
| 3 | 1617 | 2558 | 3249 | 5123 | 42.977 | 67.766 | 2.302 | 3.605 | 24.459 | 1.043 | 0.503 | 2 | 2 |
| 3 | 1691 | 2592 | 3273 | 8031 | 43.297 | 106.226 | 2.061 | 3.702 | 21.115 | 1.037 | 0.523 | 4 | 2 |
| 4 | 1217 | 1842 | 2254 | 4010 | 34.683 | 61.688 | 2.105 | 3.068 | 14.493 | 1.036 | 0.544 | 2 | 1 |
| 4 | 1305 | 1919 | 2494 | 4629 | 38.373 | 71.208 | 2.122 | 3.570 | 18.049 | 1.133 | 0.563 | 4 | 1 |
| 4 | 1240 | 1867 | 2335 | 4252 | 35.920 | 65.421 | 2.190 | 3.333 | 15.424 | 1.090 | 0.523 | 2 | 2 |
| 4 | 1221 | 1903 | 2233 | 3319 | 34.35 | 51.054 | 2.123 | 3.191 | 18.271 | 1.070 | 0.523 | 4 | 2 |
| 5 | 1621 | 2335 | 3387 | 6729 | 46.144 | 91.669 | 2.308 | 3.975 | 23.704 | 1.104 | 0.482 | 2 | 1 |
| 5 | 1838 | 3161 | 3843 | 6348 | 52.362 | 86.490 | 2.598 | 3.818 | 23.668 | 1.096 | 0.482 | 4 | 1 |
| 5 | 1755 | 2476 | 3542 | 7641 | 48.253 | 104.101 | 2.327 | 3.994 | 20.474 | 1.134 | 0.543 | 2 | 2 |
| 5 | 1535 | 2348 | 3130 | 6375 | 42.641 | 86.858 | 2.337 | 3.687 | 20.406 | 1.138 | 0.544 | 4 | 2 |
| 6 | 2143 | 3184 | 4383 | 7764 | 56.193 | 99.540 | 2.429 | 3.954 | 25.630 | 1.263 | 0.543 | 2 | 1 |
| 6 | 1684 | 3258 | 3997 | 6885 | 51.238 | 88.270 | 2.730 | 3.959 | 25.518 | 1.319 | 0.543 | 4 | 1 |
| 6 | 1870 | 3007 | 4118 | 7414 | 52.789 | 95.047 | 2.845 | 4.167 | 24.098 | 1.287 | 0.544 | 2 | 2 |
| 6 | 1844 | 3489 | 4209 | 8822 | 53.960 | 113.102 | 2.969 | 4.337 | 24.281 | 1.277 | 0.503 | 4 | 2 |
| 7 | 1724 | 2315 | 3469 | 6658 | 49.552 | 95.114 | 2.316 | 3.735 | 15.239 | 0.987 | 0.484 | 2 | 1 |
| 7 | 1885 | 2288 | 3717 | 7114 | 53.101 | 101.63 | 2.137 | 3.824 | 12.268 | 1.014 | 0.483 | 4 | 1 |
| 7 | 1717 | 2525 | 3496 | 5744 | 49.945 | 82.054 | 2.339 | 3.550 | 19.167 | 1.006 | 0.483 | 2 | 2 |
| 7 | 1993 | 2931 | 4214 | 9250 | 60.204 | 132.146 | 2.399 | 4.216 | 14.414 | 1.027 | 0.463 | 4 | 2 |
| 8 | 1291 | 1991 | 2553 | 4340 | 38.738 | 65.862 | 2.218 | 3.458 | 15.748 | 1.129 | 0.523 | 2 | 1 |
| 8 | 1187 | 2196 | 2099 | 3931 | 31.854 | 59.645 | 1.983 | 3.080 | 14.067 | 1.080 | 0.564 | 4 | 1 |
| 8 | 1094 | 1994 | 1878 | 3265 | 28.502 | 49.552 | 2.054 | 2.969 | 12.789 | 1.062 | 0.584 | 2 | 2 |
| 8 | 1127 | 2404 | 1951 | 3043 | 29.601 | 46.181 | 2.024 | 2.947 | 21.521 | 1.100 | 0.543 | 4 | 2 |
| 9 | 2149 | 3136 | 5211 | 9595 | 62.777 | 115.600 | 2.754 | 4.224 | 45.727 | 1.428 | 0.563 | 2 | 1 |
| 9 | 1959 | 3832 | 5030 | 8453 | 60.598 | 101.840 | 2.951 | 4.514 | 10.386 | 1.538 | 0.604 | 4 | 1 |
| 9 | 2027 | 3419 | 5013 | 10413 | 60.395 | 125.459 | 2.777 | 4.582 | 12.763 | 1.492 | 0.603 | 2 | 2 |
| 9 | 1922 | 3057 | 4827 | 8847 | 58.159 | 106.596 | 3.000 | 4.576 | 17.433 | 1.500 | 0.564 | 4 | 2 |
| 10 | 1527 | 2181 | 3317 | 7698 | 45.945 | 106.614 | 2.396 | 4.123 | 11.470 | 1.257 | 0.565 | 2 | 1 |
| 10 | 1646 | 2255 | 3484 | 6943 | 48.253 | 96.161 | 2.676 | 4.155 | 10.184 | 1.307 | 0.584 | 4 | 1 |
| 10 | 1486 | 2340 | 3125 | 5800 | 43.286 | 80.334 | 2.336 | 4.018 | 13.295 | 1.217 | 0.564 | 2 | 2 |
| 10 | 1729 | 2442 | 3546 | 5910 | 49.120 | 81.857 | 2.533 | 3.891 | 10.678 | 1.205 | 0.563 | 4 | 2 |
